# The Effect of a Four-Month Training Program on Body Fat and Pulmonary Parameters of Young Soccer Players

**Published:** 2019-02

**Authors:** Sami SERMAXHAJ

**Affiliations:** Department of Physical Culture, Faculty for Sport and Physical Education, University of Montenegro, Podgorica, Montenegro

## Dear Editor-in-Chief

Excess body fat has serious consequences for health. It is associated with high levels of LDL (“bad”) cholesterol and triglycerides and low levels of HDL (“good”) cholesterol. It impairs the body’s responsiveness to insulin, raising blood sugar and insulin levels. Excess body fat contributes to major causes of death and disability, including heart attacks, strokes, high blood pressure, cancer, diabetes, osteoarthritis, fatty liver and depression (1). All of this can be prevented by playing sports, such as soccer. Soccer not only helps reduce body fat, but it also has positive effects on pulmonary parameters such as forced vital capacity (FVC) and forced expiratory volume (FEV1).

Regular physical activity and exercise improve quality of life, whether you are healthy or have a lung condition (2). The aim of this research was to determine the effect of soccer training on body fat and pulmonary parameters. The research was conducted on 20 players in the age range 11–13 yr. Before participating in the study, participants underwent a medical checkup at the Sports Medicine Center in Pristina. The checkup cleared all participants for participating in the study.

In accordance with the Declaration of Helsinki, the local university ethics committee approved the study (Universe College Ethics Committee; Professor Jeton Havolli; Protocol Number: FCP 11/10/2016-2) and participants were informed of its goal and procedures and signed written consent. The initial testing took place prior to the start of the preseason, whereas the final testing was performed after four months of training. During the period between Aug and Nov 2015 exercised three times a week (in total, undertaking 48 training sessions) as part of the regular soccer training program in the Football School of Ramiz Sadiku Club in Pristina. Players were involved in the regular soccer training sessions and competed in the Kosovo elite soccer league during the season’s first macrocycle (preparation and competition) from Aug 1 through Dec 1.

Training was designed following in line with the literature and relevant bodies’ recommendations (3, 4). Training programs were based on four components: conditional (CO), technical (TE), tactical (TA) and mental (ME) component. Training sessions comprised general and specific warm-ups (20 min), the main part (35–40 min) and a cool-down (10-min running recovery). Body height and composition (total, lean and fat mass) were measured sequentially with a Martin anthropometer and a specific analyzer (InBody 720, InBody, Seoul Korea), while FVC and FVE1 were measured using a spirometer (BTL-OB MT Plus ECG).

From the collected results, a regular training program has an important statistical impact on the decrement of body fat and the increment of FVC and FEV1. All the variables showed statistically important differences between the initial and final measurements. Lean mass increased by 1.15 kg or 1.81%, body fat decreased by 0.77 kg or 3.1%, FVC increased by 0.73 and FVE1 increased by 0.39 (Table 1).

**Table 1:** Effects of the training program on lean mass, fat mass, forced vital capacity (FVC) and forced expiratory volume (FVE1)

***Variable***	***U13-Pre (M±SD)***	***U13-Post (M±SD)***	***t***	***Sig.***
Height (cm)	153.81±7.80	156.15±8.06	−5.949	0.000
Weight (kg)	42.36±7.29	43.25±7.50	−2.513	0.033
Lean mass (kg)	19.95±3.77	21.10±4.11	−5.612	0.000
Lean mass (%)	46.99±5.33	48.80±5.00	−2.598	0.029
Fat mass (kg)	5.41±5.07	4.64±4.62	1.596	0.045
Fat mass (%)	12.30±9.04	9.20±5.86	1.762	0.042
FVC	2.67±0.73	3.38±0.60	−2.436	0.038
FVE1	2.23±0.39	2.97±0.60	−3.535	0.006

**Note:** Mean=arithmetic mean, SD=Standard Deviation, *t*-test, sig*.* values, bold=significant values

Melchiori et al. showed how a training program for soccer players can significantly improve body composition in terms of overall (−1.6 kg) and fat (−2.8 kg) mass (5). Body composition was revealed to be different in soccer playing boys with two years of training experience (U11, U13, U15) compared with boys who did not play soccer (6). Regular, strong and long-time exercise, in addition to the character of training and competition, produces a positive effect on the lung by increasing pulmonary capacity, thereby increasing the lung functioning (7). A soccer training season provided balanced improvements in both anthropometric (body height, mass and fat) and performance variables (Yo-Yo intermittent recovery test level 1, countermovement jump, squat jump, five-jump test, and 10-m and 30-m sprints) in young players (8).

Therefore, the practice of soccer has positive effects on body composition (lean mass, body fat) and pulmonary parameters. By reducing body fat, the risk of being obese and developing various diseases is decreased. We recommend parents to take their children to soccer training in order to provide their children with the opportunity for healthy growth and development.
